# Unraveling the effects of maternal breastfeeding duration and exclusive breast milk on children’s cognitive abilities in early childhood

**DOI:** 10.3389/fpubh.2023.1225719

**Published:** 2023-12-01

**Authors:** Gabrielle Garon-Carrier, Gabriel Arantes Tiraboschi, Jonathan Y. Bernard, Célia Matte-Gagné, Angélique Laurent, Annie Lemieux, Caroline Fitzpatrick

**Affiliations:** ^1^Département de Psychoéducation, Université de Sherbrooke, Sherbrooke, QC, Canada; ^2^Département d’enseignement au préscolaire et primaire, Université de Sherbrooke, Sherbrooke, QC, Canada; ^3^Inserm, INRAE, Centre for Research in Epidemiology and Statistics (CRESS)Université Paris Cité and Université Sorbonne Paris Nord, Paris, France; ^4^École de psychologie, Université Laval, Québec, QC, Canada; ^5^Department of childhood education, University of Johannesburg, Johannesburg, South Africa

**Keywords:** breastfeeding, breast milk, formula, memory-span, math skills, longitudinal design

## Abstract

**Background:**

This study investigated the putative associations between mothers’ use of exclusive breast milk and the duration of breastfeeding with child cognitive development.

**Methods:**

This study is based on 2,210 Canadian families with children assessed longitudinally from age 4 to 7 years on their memory-span and math skills. These cognitive abilities were measured with standardized tasks. Breastfeeding practices were collected via maternal reports. We applied propensity scores to control the social selection bias for breastfeeding.

**Results:**

Results adjusted for propensity scores and sample weight revealed no significant differences between non-breastfed children with those being non-exclusively breastfed for 5 months or less, and with children being exclusively breastfed for 9.2 months on average, on their early math skills and memory-span. We found that children who were non-exclusively breastfed for 6.8 months on average had a slightly higher levels of memory-span at age 4 than children who were never breastfed, and this small but significant difference lasted up to age 7.

**Conclusion:**

Our findings suggest no significant differences between children being exclusively breastfed and those fed with formula on their early math skills and memory-span. The encouragement of breastfeeding to promote child cognitive school readiness may, in some case (non-exclusive breastfeeding for more than 5 months), show a small but long-lasting advantage in early memory-span.

## Introduction

Breastfeeding and human milk are considered the normative standards for infant feeding and nutrition. It has several beneficial effects including nutrition and growth, fostering immune-microbiome interplay, promoting mother–child interaction, and improving neurobehavioral outcomes, especially among premature and very low birth weight children (1, 2). Breastfeeding for 3 months or more has been determined as an adequate period according to guidelines on allergy prevention and nutrition (1). However, the World Health Organization and UNICEF (3) suggests that exclusive breastfeeding for at least 6 months is necessary to observe early cognitive gains in breastfed children.

Multiple possible mechanisms can explain the effect of breastfeeding on children’s health and cognitive development. According to the nutrient hypothesis, the docosahexaenoic (DHA) and arachidonic acids found in breast milk are involved in neural maturation, which would enhance the development of cognitive abilities such as problem-solving and memory-span (4–6). This mechanism has been shown in rats, where deficiency of DHA during lactation resulted in poor memory retention during learning tasks, whereas DHA supplementation had the reverse effect (7). Another potential mechanism for the development of cognitive abilities is the mother-infant physiological proximity during breastfeeding. According to this hypothesis, early skin-to-skin contact during breastfeeding would accelerate neuromaturation and thus, the development of cognitive abilities. One additional explanation is that mothers breastfeeding their infant are also more likely to provide a cognitively stimulating environment. Several studies revealed a negligible relationship between breastfeeding and child cognitive functioning after adjusting for maternal and home environment (8–10). This later explanation suggests controlling for the quality of the home environment as breastfeeding may be a proxy for parenting (11, 12).

Yet, despite these possible mechanisms, evidence that breastfeeding promotes child cognitive development is mixed. Some studies support a small but significant direct effect of breastfeeding on child cognitive abilities (2, 13–21). One cluster-randomized trial study found evidence that prolonged and exclusive breastfeeding (intervention group) improved children’s IQ scores at age 6.5 years in comparison with controls (18). One observational US study also revealed that breastfeeding remained significantly associated with the child cognitive functioning measured with the Bayley Scales of Infant Development (BSID-II) at age 2 years, even after matching children being breastfed to those not being breastfed (14). In contrast, other studies found little to no relationship between breastfeeding practices and child cognitive development (8, 22–24). In a US national cohort study of 5,475 children of normal birth weight, ever being breastfed was associated with almost a 5-point higher child IQ, but this effect disappeared after adjustment for confounders (23). Another study in the US found no significant effects of any breastfeeding, breastfeeding duration, or exclusive breastfeeding on child executive function during mid-childhood (22). Another study revealed no significant association between breastfeeding for 3 months or longer and child math and reading skills at age 4 (8).

One possible explanation for these mixed findings is that previous studies have not adequately disentangle the duration of breastfeeding and whether children were exclusively and/or predominantly breastfed (20). Previous studies examining how breastfeeding practices are associated with children’s cognitive development have also been limited by their study design, with most studies not controlling the selection bias for breastfeeding (2, 8, 13, 16, 19–22). Over the past decade, the use of statistical methods, such as propensity score weighting and the use of instrumental variables have strengthened the possibility of drawing causal inferences on the long-term cognitive outcomes of breastfeeding.

Another limitation is that only a few studies have adopted a longitudinal design with repeated measures of children’s cognitive abilities (13, 16, 20, 21, 24). One longitudinal study found that having been breastfed (yes/no) was associated with a small but significant advantage in IQ at age 2 in girls but was not associated with IQ growth from ages 2 to 16 (21). This study, however, did not include measures of breastfeeding duration or breastfeeding exclusivity and did not control for the selection bias for breastfeeding. Furthermore, few studies have examined outcomes in the preschool and early school years (2, 24). Examining the effect of breastfeeding on child cognitive skills is particularly salient during the transition to school entry, when cognitive skills become important determinants of school readiness and later academic achievement. To our knowledge, only one study conducted in Ireland examined the association between breastfeeding and child cognitive abilities (problem-solving, expressive vocabulary) repeatedly at ages 3 and 5 years while controlling for selection bias (24). However, they did not control for parenting and home environment factors. This study also disentangled the duration of breastfeeding (1 month, 2–6 months, 6 months, or more) and the breastfeeding practices: full (exclusive or almost exclusive) and partial breastfeeding, in comparison to never being breastfed. Interestingly, children who were fully breastfed for 6 months or more had higher problem-solving scores at age 3 years in comparison to children who were never breastfed. However, this association was no longer statistically significant at age 5 and did not remain significant at age 3 after adjustment for multiple testing. These findings warrant replication.

### Objectives

This study aims to test the effects of breastfeeding on children’s cognitive development (early math skills and memory-span) during the transition to school entry. Specifically, we examine how the duration of breastfeeding and exclusive use of breast milk are longitudinally associated with children’s early memory-span and math skills (including problem-solving), while controlling the selection bias for breastfeeding due to child, maternal/family, and demographic confounding variables. By doing so, we attempt to disentangle some of the various mechanisms potentially explaining such association. Infants exclusively breastfed and showing the highest levels of memory-span scores and early math skills would provide support to the nutrient hypothesis. To dismiss the hypothesis that breastfeeding mothers also provide a more cognitively stimulating environment, our analyses were adjusted to account for parenting practices.

## Methods

### Sample and design

Participants were from the Quebec Longitudinal Study of Child Development (QLSCD), an ongoing longitudinal population-based study aimed at understanding the impact of early experiences on later school success (25). Families were recruited through the Quebec Master Birth Registry of the Ministry of Health and Social Services to be representative of children born in 1997–98 in Quebec, Canada. For practical reasons, data were not collected on children living on Cree or Inuit territories, in Indian reserves, and in northern Quebec. A three-stage sampling design based on living area and birth rate was used. Territories were first divided into regions, which were then divided into second-stage units composed of one or two county regional municipalities, and then further divided in third-stage units according to the number of births in 1996. All selected infants were born after October 1, 1997 to ensure that they entered school the same year. Families were excluded if mothers could not speak French or English, and if babies were born before 24 weeks or after 42 weeks of gestation. A sample of 2,940 families with newborns was initially identified. Selected families that could be located (*N* = 2,675) were approached by mail and phone. Of those, 2,223 families were first visited when the child was 5 months old (83%) and 2,210 were followed longitudinally and were assessed every year up to age 23. Ethics approval was obtained from the Direction Santé Québec of the Institut de la statistique du Québec and the Faculty of Medicine of the Université de Montréal. The respondent provided consent and voluntarily responded to this survey. The analytic sample for this study included families for whom information was available about the duration of breastfeeding and the use of exclusive breast milk when the baby was 5 months old (*N* = 2,120).

### Measures

#### Breastfeeding practices

Breastfeeding practices were measured using two items reported by the mother when the baby was age 5 months: “Did you breastfeed your baby? (1 = yes, and I am continuing to do so; 2 = yes, but I have since ceased to do so; 3 = no, I never did). Mothers who reported breastfeeding their child and continuing to do so were considered as breastfeeding for more than 5 months, while mothers reporting that they ceased to breastfeed were grouped as breastfeeding for less than 5 months. Mothers that breastfed their infants for more than 5 months were also asked the following question: “Did your baby drink anything other than just breast milk? (yes/no).” At the 17 months interview, mothers breastfeeding their infants also reported how old was their infant (in months) when they ceased breastfeeding.

Four groups of mothers were derived from these items: (1) non-breastfeeding group (commercial milk only, *n* = 600, 28.3%), (2) non-exclusive breastfeeding for 5 months or less (*n* = 809, 38.2%), (3) non-exclusive breastfeeding for more than 5 months (*n* = 356, 16.8%), and (4) exclusive breastfeeding (breast milk only) for more than 5 months (*n* = 355, 16.7%). None of the mothers breastfeeding for 5 months or less used exclusive breast milk.

#### Child cognitive abilities

Children’s early math skills were measured at ages 4, 5, and 6 years with the Number Knowledge Test (26–29). The Number Knowledge Test was developed to document children’s understanding of whole numbers and basic operations, and as a tool for teachers to identify children with mathematic difficulties (30). This test has four levels of complexity (from 0 to 3). Each level of the test reflects a current developmental stage of children’s number knowledge comprehension (30, 31). The baseline and first levels of the Number Knowledge Test were administered at ages 4, 5, and 6. Except for the low reliability at age 5 (Cronbach’s α = 0.55), the internal consistency of the Number Knowledge Test in our sample was found to be adequate (α = 0.68 at age 4, 0.92 at age 6); and the test–retest stability was high across all time points (Pearson’s r = 0.74 between ages 4 and 5 and r = 0.92 between ages 5 and 6).

Children were also assessed on their memory-span at ages 4, 5, 6, and 7 years with the Visually Cued Recall task (32), a reliable measure (α = 0.95 in our sample) of the child’s incremental capacity to encode visual items and to recall the spatial locations of the items after a short delay. In each trial, a research assistant showed a cardboard with pictures of 12–18 objects to the child. The research assistant pointed to a certain number of objects and asked the child to remember them. The research assistant then flipped the cardboard for a short delay. When flipped back, the child was cued to identify the objects pointed to previously. The number of objects to remember increased after each trial, up to 12 different levels of difficulty. The test ended when the child made two errors on two consecutive levels. The final score consists of the highest level reached by the child.

#### Covariates

Covariates were selected as controls for empirical and theoretical reasons (22–24). When children were 5 months old, the person most knowledgeable about the child (99.0% were mothers) provided data on the household income (<30 K CAD/year vs. higher income), maternal education (university diploma vs. no university diploma), if the mother was an immigrant (yes/no), maternal age, and family composition (single-parent, two-parent, or stepfamily). Birth weight (<2,500 g) and developmental/stunted growth (<10^th^ centile) of the child were derived from the birth medical registry. The mothers also reported if they worked since pregnancy (yes/no).

Maternal smoking during pregnancy was coded present if the mother had smoked at least one cigarette/day while pregnant. Prenatal alcohol exposure was coded as 0 = never, 1 = having drunk alcohol less than 3 times/month. Symptoms of maternal depression in the last week were rated at the 5 months interview with the 12-item version (α = 0.85) of the Center for Epidemiologic Studies Depression Scale (33). Item responses ranged from 0 (none) to 3 (all the time), and the total scores were then rescaled on a 10-point scale. Two dimensions of parenting were also reported by the mother in the Parental Cognitions and Conduct toward the Infant Scale (34): overprotection (e.g., keeping the child close most of the time; 5 items, α = 0.68) and perceived parental impact (e.g., thinks his/her parenting affects the emotional development of the child; 5 items, α = 0.71).

### Procedure

A trained research assistant administered the Visually Cued Recall task test following a standard procedure in a face-to-face interview. The Number Knowledge Test was orally administered one-on-one by a trained research assistant at school or at home. Breastfeeding practices and the various child, maternal/family, and demographic confounding variables were reported by the mothers at the 5-months interview.

### Analytical strategy

**Figure 1:**
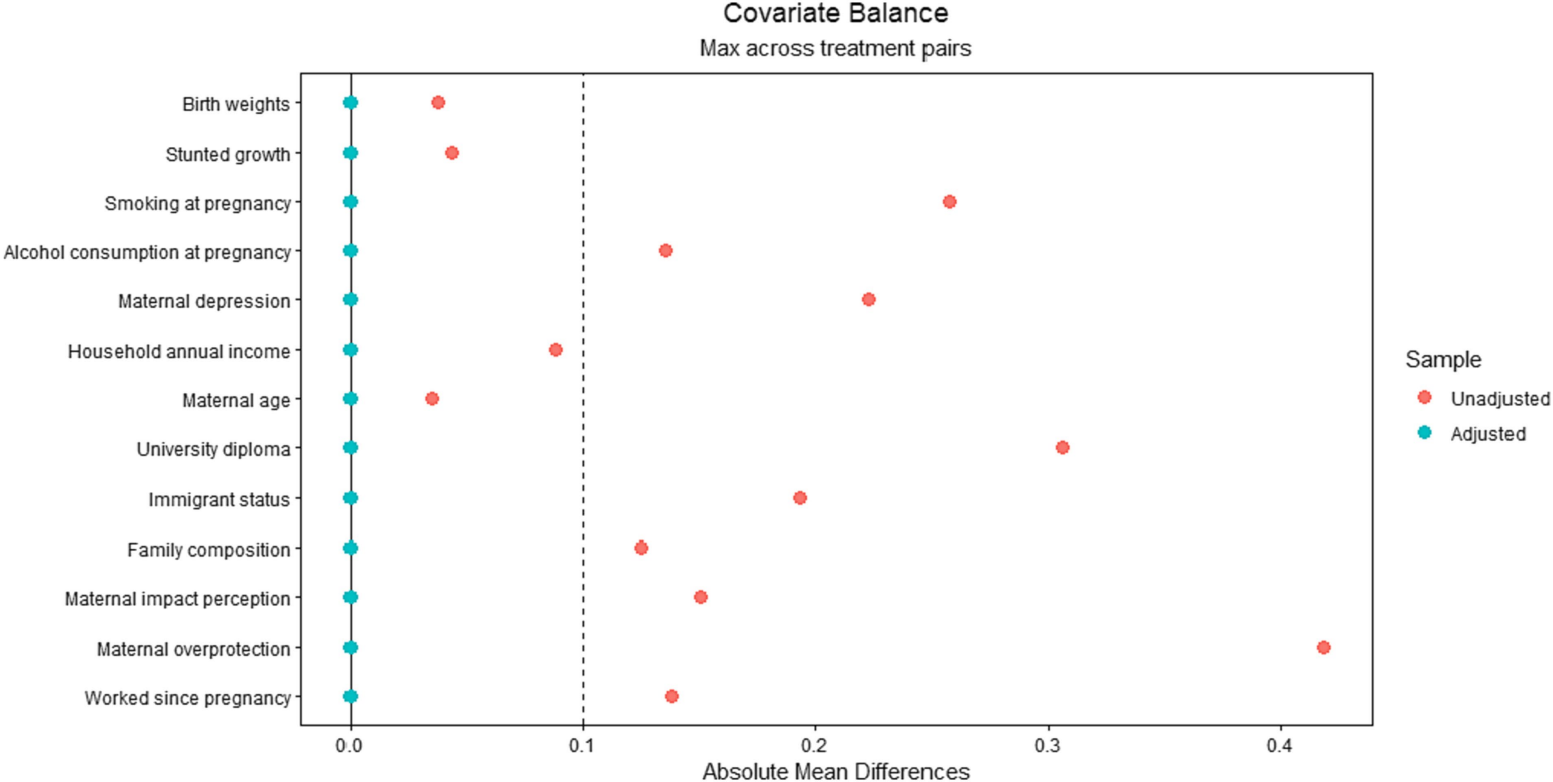
The figure displays the average standardized mean difference between groups for all the covariates used in the estimation of the propensity score weights. The orange dots represent the differences among groups on each covariate before applying the propensity score weights, and the blue dots represents the differences after application of the propensity score weights.

We performed covariate balancing propensity score (CBPS) weighting in R to increase comparability across the four groups of mothers (35). Indeed, breastfeeding does not occur at random, since mothers breastfeeding their babies are not similar to non-breastfeeding mothers regarding important covariates. This procedure reduces the selection bias for breastfeeding. CBPS performed multinomial regression to estimate the associations between the covariates and the four groups of mothers and generates a propensity score for each observation. The propensity score estimates the predicted probability of group membership from all observed covariates. Once estimated, we conducted a balancing test to ensure the quality of weighting (36). The balancing test showed that all covariates had a standardized mean difference less than |0.10| after CBPS, indicating that group differences were minimal (37) (see Figure 1).

After applying the CBPS, we conducted Latent Growth Modeling (LGM) to investigate how the groups based on the duration of breastfeeding and exclusive breast milk were longitudinally associated with changes in children’s math ability and memory-span across time. LGM is a special class of Confirmatory Factor Analysis that estimate systematic change or growth over a period of time (also called trajectory), and the inter-individual variability in this change (38). The trajectory can be of various shapes (linear, quadratic, cubic). First, for each child’s cognitive outcome, an unconditional (baseline) model was estimated to determine the average trajectory of early math skills and memory-span, using the maximum-likelihood technique for continuous and normally distributed data. Second, conditional growth models (i.e., including predictors) were performed to predict the developmental trajectory for each child outcome from groups of breastfeeding mothers. The goodness of fit of these models were determined with a root means square error of approximation (RMSEA) < 0.08 (39), a comparative fit index (CFI) > 0.90 (40), and a value of chi-square small enough not to reach the significance threshold (41).

The LGM was performed in Mplus (42) using the full information maximum likelihood to handle missing outcome data. The analyses were adjusted for the propensity scores and for sample weight from the QLSCD, which ensures that the sample remains representative of the Quebec population. It was also adjusted for the covariates as additional controls. This allowed us to eliminate the selection bias for breastfeeding while also removing the contribution of these covariates to the predicted outcomes (43). Code for the analysis is available by emailing the corresponding author.

### Pattern of missing data

The average proportion of missing data across covariates was 1.5%. Considering the low proportion of missing data, missing data were replaced by the mean for continuous variables, the median for ordinal variables, or the mode for categorical variables. The proportion of missing data on child early math skills and memory-span was on average 12.0 and 7.4% per year, respectively. According to Little’s test, the overall pattern of missingness significantly deviates from a pattern of data that is missing completely at random (*χ*^2^ = 244.42, df = 172, *p* = 0.000). The pattern of missingness was most likely at random. A series of t-tests and chi-square revealed that children with missing scores on the number knowledge and memory-span tests tended to be from a lower socioeconomic background, from immigrant and single mothers, younger than 20 years old, with no university diploma, and smoking and drinking during pregnancy. We statistically controlled for these variables in our analyses.

## Results

Mothers who never breastfed used commercial milk exclusively. Mothers breastfeeding for 5 months or less stopped breastfeeding when their babies were on average 2.1 months (SD = 1.68). The group of mothers with non-exclusive breastfeeding for more than 5 months introduced commercial milk when their babies were 2.9 months (SD = 0.46) and stopped breastfeeding when their babies were on average 6.8 months old (SD = 1.83). The group of mothers with exclusive breastfeeding for more than 5 months stopped breastfeeding when their infants were 9.2 months on average (SD = 2.73). Descriptive statistics of confounding variables and child cognitive abilities prior to applying CBPS are shown for each group in Table 1.

**Table 1:** Child, maternal and family-wide factors associated with breastfeeding when the child was 5 months-old (*N* = 2,120).

	Breastfeeding > 5 months		Breastfeeding < or = 5 months	No breastfeeding
	Exclusive breast milk (*n* = 355, 16.7%)	Non-exclusive breast milk (*n* = 356, 16.8%)	Non-exclusive breast milk (*n* = 809, 38.2%)	Exclusive formula (*n* = 600, 28.3%)
Child characteristics				
At birth				
Sex of the child (female, *n* = 1,040)	49.9%	47.8%	49.4%	48.8%
Low birth weight (<2,500 g, *n* = 73)	2.8%	3.9%	2.3%	5.0%
Stunted growth (yes = 172)	7.0%	5.6%	8.5%	9.7%
Maternal characteristics				
At birth				
Maternal age (< 20 years old, *n* = 59)	1.4%	0.8%	3.7%	3.5%
Immigrant status (yes, *n* = 253)	18.3%	19.1%	10.6%	5.7%
University diploma (yes, *n* = 566)	38.0%	44.1%	25.2%	11.7%
Alcohol consumption at pregnancy (yes, *n* = 752)	42.5%	38.8%	38.3%	25.5%
Ever smoked during pregnancy (yes, *n* = 533)	15.2%	12.6%	25.8%	37.5%
At 5 months				
Maternal depression score^†^	1.27 (1.26)	1.22 (1.29)	1.44 (1.32)	1.53 (1.42)
Maternal impact perception score^†^	8.46 (1.85)	8.56 (1.71)	8.38 (1.84)	8.17 (1.90)
Maternal overprotection score^†^	5.35 (2.30)	4.78 (2.23)	4.37 (2.10)	4.82 (2.15)
Worked since pregnancy (yes, *n* = 422)	9.9%	16.6%	24.9%	21.0%
Family-wide factors				
At 5 months				
Household annual income (< 30 K, *n* = 483)	25.0%	17.1%	20.4%	28.0%
Family composition (single parent, *n* = 406)	17.2%	13.8%	17.6%	25.7%

Data are courtesy of the Quebec Institute of Statistics.

† Indicates continuous variables.

### Early math skills and memory-span developmental growth

The unconditional LGM models yield significant intercept and slope for both outcomes, indicating progressive growth over time in early math skills and memory-span. Early math skills revealed a linear growth while the memory-span trajectory had a quadratic shape. Developmental trajectories of early math skills and memory-span are displayed in Supplementary Figures S1, S2. The unconditional LGM for early math skills had an acceptable fit as evidenced by the non-significant chi-square (*χ*^2^ = 0.538, *p* = 0.463), the RMSEA = 0.000 [0.000; 0.055], and the CFI = 1.00. The unconditional LGM for memory-span also had an adequate fit (*χ*^2^ = 3.88, *p* = 0.143; RMSEA = 0.022 [0.000; 0.055]; CFI = 0.989).

### Associations of breastfeeding with early math skills and memory-span

We next examined the extent to which the groups of maternal breastfeeding predicted the initial level and the growth in early math skills and memory-span, once adjusted for the propensity scores and for sample weight, and while controlling for birth weights, stunted growth, smoking and alcohol drinking during pregnancy, maternal depression, household income, maternal age, maternal education, immigrant status, family composition, maternal perception of impact and overprotection, and working since pregnancy. Despite the adequate fit of the conditional model (*χ*^2^ = 16.87, *p* = 0.462; RMSEA = 0.000 [0.000; 0.021]; CFI = 1.00), our findings revealed no significant associations between the groups of breastfeeding mothers (vs. non-breastfeeding group) and children’s early math skills’ intercept and slope. Results are shown in Table 2.

**Table 2:** Association between breastfeeding and early math skills (standardized estimates).

	Intercept (R^2^ = 0.152)					Slope (R^2^ = 0.039)				
	Estimate	SE	CI	*p*	Beta	Estimate	SE	CI	*p*	Beta
Mean	4.85	0.675	3.53; 6.17	0.000		3.16	0.428	2.32; 4.00	0.000	
Variance	7.17	1.02	5.15; 9.18	0.000		2.20	0.407	1.40; 3.00	0.000	
Predictors										
Breastfeeding <5 months	−0.137	0.271	−0.67; 0.39	0.613	−0.020	0.051	0.162	−0.26; 0.36	0.750	0.015
Non-exclusive breastfeeding >5 months^a^	0.539	0.321	−0.91; 1.17	0.094	0.082	−0.046	0.192	−0.42; 0.33	0.811	−0.013
Exclusive breastfeeding >5 months^a^	0.049	0.330	−0.59; 0.69	0.881	0.007	0.067	0.210	−0.34; 0.47	0.748	0.019
Covariates										
Birth weights	0.794	0.784	−0.74; 2.33	0.311	0.052	−0.243	0.540	−1.30; 0.81	0.653	−0.031
Stunted growth	−0.733	0.403	−1.52; 0.05	0.069	−0.071	−0.458	0.305	−1.05; 0.14	0.133	−0.085
Smoking during pregnancy	−0.152	0.266	−0.67; 0.37	0.569	−0.023	0.258	0.167	−0.06; 0.58	0.122	0.074
Alcohol during pregnancy	−0.130	0.232	−0.58; 0.32	0.576	−0.022	−0.020	0.139	−0.29; 0.25	0.883	−0.007
Maternal depression	0.088	0.085	−0.07; 0.25	0.297	0.042	−0.028	0.060	−0.14; 0.08	0.636	−0.026
Household income	**−0.726**	**0.291**	**−1.29; 0.16**	**0.012**	**−0.101**	−0.115	0.181	−0.46; 0.23	0.523	−0.031
Maternal age	−0.817	0.670	−2.13; 0.49	0.223	−0.045	0.840	0.469	−0.08; 1.76	0.074	0.089
University degree	**1.602**	**0.269**	**1.07; 2.13**	**0.000**	**0.246**	−0.262	0.161	−0.57; 0.05	0.104	−0.077
Immigrant status	0.337	0.487	−0.62; 1.29	0.489	0.034	−0.041	0.290	−0.61; 0.52	0.887	−0.008
Intact family	**−0.734**	**0.275**	**−1.27; −0.19**	**0.008**	**−0.097**	0.138	0.179	−0.21; 0.49	0.441	0.035
Perception of impact	0.114	0.059	−0.002; 0.22	0.054	0.069	0.066	0.039	−0.00; 0.14	0.086	0.077
Overprotection	−0.065	0.052	−0.17; 0.03	0.210	−0.049	0.025	0.034	−0.04; 0.09	0.456	0.036
Worked since pregnancy	**−0.553**	**0.271**	**−1.09; −0.02**	**0.041**	**−0.077**	−0.043	0.171	−0.37; 0.29	0.803	−0.011

SE: standard error; bold values denote statistical significance based on the confidence interval (CI). This analysis was adjusted for the propensity scores and for sample weight from the QLSCD. As indicated in the table, it was also adjusted for the following covariates: birth weights, stunted growth, smoking and alcohol during pregnancy, maternal depression, household income, maternal age, maternal education, immigrant status, family composition, maternal perception of impact and overprotection, and working since pregnancy.

a In comparison to the non-breastfeeding group (commercial milk only).

The conditional model also revealed an acceptable fit in predicting memory-span (*χ*^2^ = 23.58, *p* = 0.169; RMSEA = 0.013 [0.000; 0.025]; CFI = 0.981). Results are shown in Table 3. In comparison to the non-breastfeeding group, the group of mothers non-exclusively breastfeeding for more than 5 months was significantly associated with the initial level in child memory-span. Children who were non-exclusively breastfed for more than 5 months had higher levels of memory-span at age 4 than children who were never breastfed. This small (beta = 0.08) but significant difference between the two groups was maintained over time up to age 7.

**Table 3:** Association between breastfeeding and memory-span (standardized estimates).

	Intercept (R^2^ = 0.095)					Slope (R^2^ = 0.071)					Quadratic (R^2^ = 0.209)				
	Estimate	SE	CI	*p*	Beta	Estimate	SE	CI	*p*	Beta	Estimate	SE	CI	*p*	Beta
Mean	2.90	0.349	2.21; 3.58	0.000		1.03	0.559	−0.06; 2.12	0.065		−0.063	0.187	−0.42; 0.30	0.734	
Variance	3.65	0.735	2.21; 5.09	0.000		3.00	0.665	1.70; 4.31	0.000		0.100	n/a	n/a	n/a	
Predictors															
Breastfeeding <5 months^a^	0.028	0.144	−0.25; 0.31	0.847	0.006	−0.020	0.243	−0.49; 0.45	0.934	−0.005	0.003	0.079	−0.15; 0.16	0.969	0.004
Non-exclusive breastfeeding >5 months^a^	**0.367**	**0.184**	**0.01; 0.73**	**0.046**	**0.080**	−0.463	0.285	−1.02; 0.09	0.104	−0.113	0.109	0.095	−0.07; 0.29	0.250	0.134
Exclusive breastfeeding >5 months^a^	−0.254	0.171	−0.59; 0.08	0.137	−0.054	−0.222	0.307	−0.82; 0.38	0.470	−0.053	0.090	0.100	−0.10; 0.28	0.367	0.108
Covariates															
Birth weights	−0.295	0.352	−0.98; 0.39	0.402	−0.028	0.278	0.619	−0.93; 1.49	0.653	0.029	−0.066	0.204	−0.46; 0.33	0.748	−0.035
Stunted growth	**−0.512**	**0.213**	**−0.93; −0.09**	**0.016**	**−0.071**	0.104	0.343	−0.56; 0.77	0.762	0.016	0.009	0.137	−0.25; 0.27	0.948	0.007
Smoking during pregnancy	−0.151	0.141	−0.42; 0.12	0.285	−0.033	−0.190	0.260	−0.70; 0.31	0.464	−0.046	0.097	0.087	−0.07; 0.26	0.265	0.119
Alcohol during pregnancy	0.146	0.133	−0.11; 0.40	0.272	0.035	0.301	0.220	−0.13; 0.73	0.171	0.081	−0.105	0.072	−0.24; 0.03	0.148	−0.142
Mother depression	**−0.091**	**0.040**	**−0.17; −0.01**	**0.023**	**−0.062**	−0.003	0.075	−0.14; 0.14	0.972	−0.002	0.011	0.025	−0.03; 0.06	0.653	0.043
Household income	**−0.489**	**0.146**	**−0.77; −0.20**	**0.001**	**−0.099**	0.275	0.268	−0.25; 0.80	0.304	0.062	−0.055	0.092	−0.23; 0.12	0.552	−0.063
Maternal age	−0.429	0.339	−1.09; 0.23	0.207	−0.035	0.058	0.510	−0.94; 1.05	0.909	0.005	−0.052	0.160	−0.36; 0.26	0.747	−0.024
University degree	**0.687**	**0.153**	**0.38; 0.98**	**0.000**	**0.152**	**−0.505**	**0.252**	**−0.99; −0.01**	**0.045**	**−0.125**	0.111	0.081	−0.04; 0.27	0.172	0.139
Immigrant status	−0.148	0.180	−0.50; 0.20	0.411	−0.022	0.071	0.393	−0.70; 0.84	0.856	0.012	0.021	0.131	−0.23; 0.27	0.871	0.018
Intact family	0.104	0.158	−0.20; 0.41	0.509	0.020	**−0.558**	**0.279**	**−1.1; −0.01**	**0.045**	**−0.120**	**0.225**	**0.095**	**0.04; 0.41**	**0.017**	**0.245**
Perception of impact	0.033	0.031	−0.02; 0.09	0.288	0.029	**0.181**	**0.052**	**0.07; 0.28**	**0.001**	**0.177**	**−0.059**	**0.017**	**−0.09; −0.02**	**0.001**	**−0.290**
Overprotection	−0.003	0.032	−0.06; 0.06	0.927	−0.003	−0.008	0.049	−0.10; 0.08	0.872	−0.010	0.000	0.016	−0.03; 0.03	0.990	0.001
Worked since pregnancy	0.343	0.177	−0.00; 0.69	0.052	0.069	−0.116	0.287	−0.67; 0.44	0.687	−0.026	0.014	0.095	−0.17; 0.20	0.879	0.016

SE: standard error; bold values denote statistical significance based on confidence interval (CI). n/a indicates not available. This analysis was adjusted for the propensity scores and for sample weight from the QLSCD. As indicated in the table, it was also adjusted for the following covariates: birth weights, stunted growth, smoking and alcohol during pregnancy, maternal depression, household income, maternal age, maternal education, immigrant status, family composition, maternal perception of impact and overprotection, and working since pregnancy.

a In comparison to the non-breastfeeding group (commercial milk only).

## Discussion

This study investigated the putative associations between mothers’ use of exclusive breast milk and the duration of breastfeeding with children’s early memory-span and math skills. Similar to previous studies (8, 22–24), our findings revealed little to no significant differences between children being breastfed and those fed with formula (non-breastfed infants) on their early math skills and memory-span. No significant differences were found between non-breastfed children and those being non-exclusively breastfed for 5 months or less, and with children being exclusively breastfed for more than 5 months. Interestingly, children being non-exclusively breastfed for more than 5 months showed a slightly higher levels of memory-span that lasted over time compared to the non-breastfed group.

One possible explanation is that mothers non-exclusively breastfeeding for more than 5 months may benefit from greater marital support and/or from an extend social network, where the mother breastfeed her infant every time she can but the father (and/or grand-parents, educators) also have the chance to bottle-fed the infant. In turn, this social network may provide different and various stimulating interactions to the child, ensuing greater memory-span skills during the preschool years. For children being non-exclusively breastfed for more than 5 months, this small (almost negligible) but significant advantage in memory-span during early childhood could still be translated into later gains in other cognitive components (ex., executive functions) or academic skills.

This finding partially supports the need to keep breastfeeding for more than 5 months. This group of mothers stopped breastfeeding when their babies were on average 6.8 months old. However, contrary to the WHO guidelines (2003) that recommend exclusive breast milk for the first 6 months and to continue breastfeeding with complementary foods until 2 years or beyond, our results rather show that it is a mix of breast milk and formula that confer benefits on memory-span. In this study, we did not distinguish between breastfeeding and breast milk that was bottled-fed to infants. However, one recent study revealed that among infants exclusively fed with breast milk, those fed directly from the mother scored higher on several memory tasks compared to children bottle-fed of breast milk (44), suggesting that nursing infants directly at the breast may impact memory.

As we found no significant difference between non-breastfed infants and those being exclusively breastfed for at least 5 months (who kept breastfeeding up to age 9.2 months on average), our findings did not support the nutrient hypothesis in promoting child cognitive development. This hypothesis postulates that the nutrients found in breast milk (e.g., DHA, arachidonic acid) facilitate neural maturation and the development of the nervous system (4–6), improving children’s cognitive growth. Similarly, this result did not support the early skin-to-skin contact to be the main mechanism in promoting the development of cognitive abilities during breastfeeding. If so, we would have found a significant difference in children’s cognitive development between children being breastfed and those from the non-breastfed group. This study, however, cannot rule out the role of these mechanisms in promoting cognitive development, as we did not directly compare the breast milk composition with infant formulas, and we did not measure the frequency of the skin-to-skin contact nor the mother–child bonding.

A key strength of this study is that we controlled for parenting practices involved in child cognitive development. Specifically, the perception of parental impact from the mother was significantly associated with growth in memory-span. Future studies should test parenting practices as potential mediating mechanisms (8, 45).

Although not entirely supporting the WHO guidelines for promoting child cognitive development, our findings do not contradict the many health benefits afforded to infants as a result of breastfeeding (46, 47). Previous studies have shown that breastfeeding decreases the risk of being overweight during infancy (46) but not in adolescence (48), and reduced the risk of chronic diseases such as allergies and asthma (49, 50). Several studies showing the benefits of breastfeeding on child cognitive outcomes were also conducted on preterm or very low-weight infants (1, 2), suggesting that poor fetal growth moderates this association. Future studies should further explore how other perinatal risk factors, such as delivery complications, parental mental health problems, and socioeconomic adversity, may moderate the association between breastfeeding and child cognitive development. Nevertheless, our results revealed that, at the population level, exclusive breastfeeding for more than 5 months (9.7 months on average) does not translate into long-term improvement in memory-span and math skills during early childhood.

## Limitations

Despite these new insights, results should be interpreted with caution. First, information on breastfeeding was collected retrospectively when infants were 5 and 17-months old. Although the reliability of recall has been established (51), recall bias may still be present, particularly regarding the duration of full breastfeeding. Second, we could not disentangle the effect of direct breastfeeding versus expressed breast milk feeding, limiting our capacity to investigate whether the association with improved memory-span could partly be the result of skin-to-skin contact. Some studies also revealed that feeding bottled breast milk may not be biologically equivalent to direct breastfeeding. Differences have been observed for infant memory-span (44), suggesting a potential negative impact from the process of bottle feeding and/or reduced bioactivity of expressed breast milk. Future research should capture the complexity of modern feeding practices, even among exclusively breast (milk)-fed infants. Similarly, misclassification of breastfeeding exposure is also possible. Here, we considered the duration and exclusivity of breastfeeding. However, recent research suggests also distinguishing nursing at the breast from expressed breast milk, the relative proportion of breast milk from infant formula and variation in the type of formula used, perinatal feeding exposures in hospitals, and introduction of complementary foods (52). Another limitation is that we did not have information about the mother’s diet such as the frequency and the quality of the food consumption, including intake of vitamin and/or mineral supplementation. As evidence by recent systematic reviews [e.g. (53, 54)] maternal diet is reflected in the breast milk composition, which might impact the nutritional quality of the breast milk and its contribution to children’s cognitive development. Third, despite our conservative approach to address the selection bias to breastfeed and to additionally controlled for several children, maternal/family, and sociodemographic confounders, we cannot rule out the possibility that selection for breastfeeding exposure resulted from confounding variables not considered in our covariate balancing propensity score approach. For instance, we did not control for parenting practices specifically tapping into memory-span or the early math domain (ex., playing with numbers) and these practices were only indirectly linked to cognitive skills (e.g., perception of impact). Similarly, we did not control for maternal IQ as it was not collected in this cohort (8). A few studies suggest that maternal IQ accounts for a large proportion of the association between breastfeeding and cognitive outcomes (17, 23). Fourth, we did not measure every component of cognitive development. For instance, breastfeeding may not be related to early math skills but may be associated with executive functions, which is located in the prefrontal cortex, a brain area imprinted by postnatal experiences (55).

## Conclusion

In conclusion, this study found little evidence that breastfeeding is longitudinally associated with early math skills and memory-span, regardless of the duration of breastfeeding and whether it was exclusively breast milk. Breastfeeding has important health and economic benefits, and the encouragement of breastfeeding to promote child cognitive school readiness may, in some case (i.e., non-exclusive breastfeeding for more than 5 months), show a small and long-lasting advantage in early memory-span. This advantage could potentially promote the development of other cognitive skills or still manifest later in life, but this has not been tested in the current study.

## Data availability statement

The data analyzed in this study is subject to licenses/restrictions. Requests for data access should be directed to https://www.jesuisjeserai.stat.gouv.qc.ca/informations_chercheurs/acces_an.html.

## Ethics statement

The study involving humans were approved by the Direction Santé Québec of the Institut de la statistique du Québec and the Faculty of Medicine of the Université de Montréal. The study were conducted in accordance with the local legislation and institutional requirements. Written informed consent for participation in this study was provided by the participants’ legal guardians/next of kin.

## Author contributions

GG-C conceived the project, conducted and oversaw all aspects of the analyses, and wrote the paper. GT conducted the analyses, contributed to the interpretation, and reviewed the manuscript. JB, CM-G, ALa, and CF contributed to the interpretation, and reviewed the manuscript. ALe conducted the analyses. All authors contributed to the article and approved the submitted version.
