# Supplementary material for: Unraveling the effects of maternal breastfeeding duration and exclusive breast milk on children’s cognitive abilities in early childhood
Source: Front Public Health. 2023 Dec 1;11:1225719. doi: 10.3389/fpubh.2023.1225719 (PMC10722166; doi:10.3389/fpubh.2023.1225719)
Supplement: Supplementary file 1 [file Table_1.DOCX]

**Supplementary materials**

**Unraveling the effects of maternal breastfeeding duration and exclusive breast milk on children’s**

**cognitive abilities in early childhood**


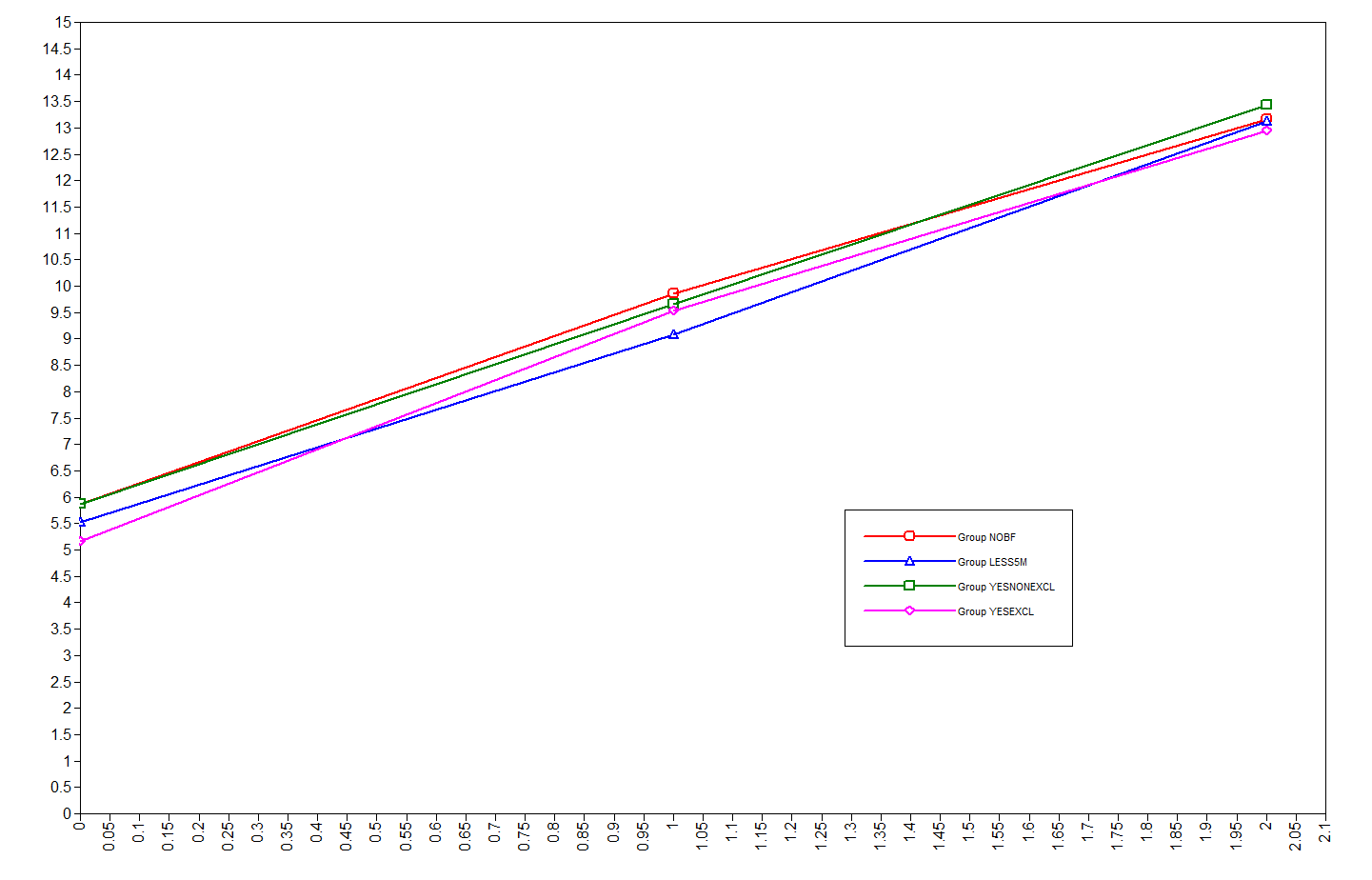


Figure S1. The figure displays the intercept and growth in early math skills from ages 4 to 6. The red line indicates non-breastfed children (n= 600, 28.3%). The blue line indicates children non-exclusively breastfed for 5 months or less (n= 809, 38.2%). The green line indicates children non-exclusively breastfed for more than 5 months (n= 356, 16.8%). The pink line indicates children exclusively breastfed for more than 5 months (n= 355, 16.7%). Data courtesy of the Quebec Institute of Statistics.


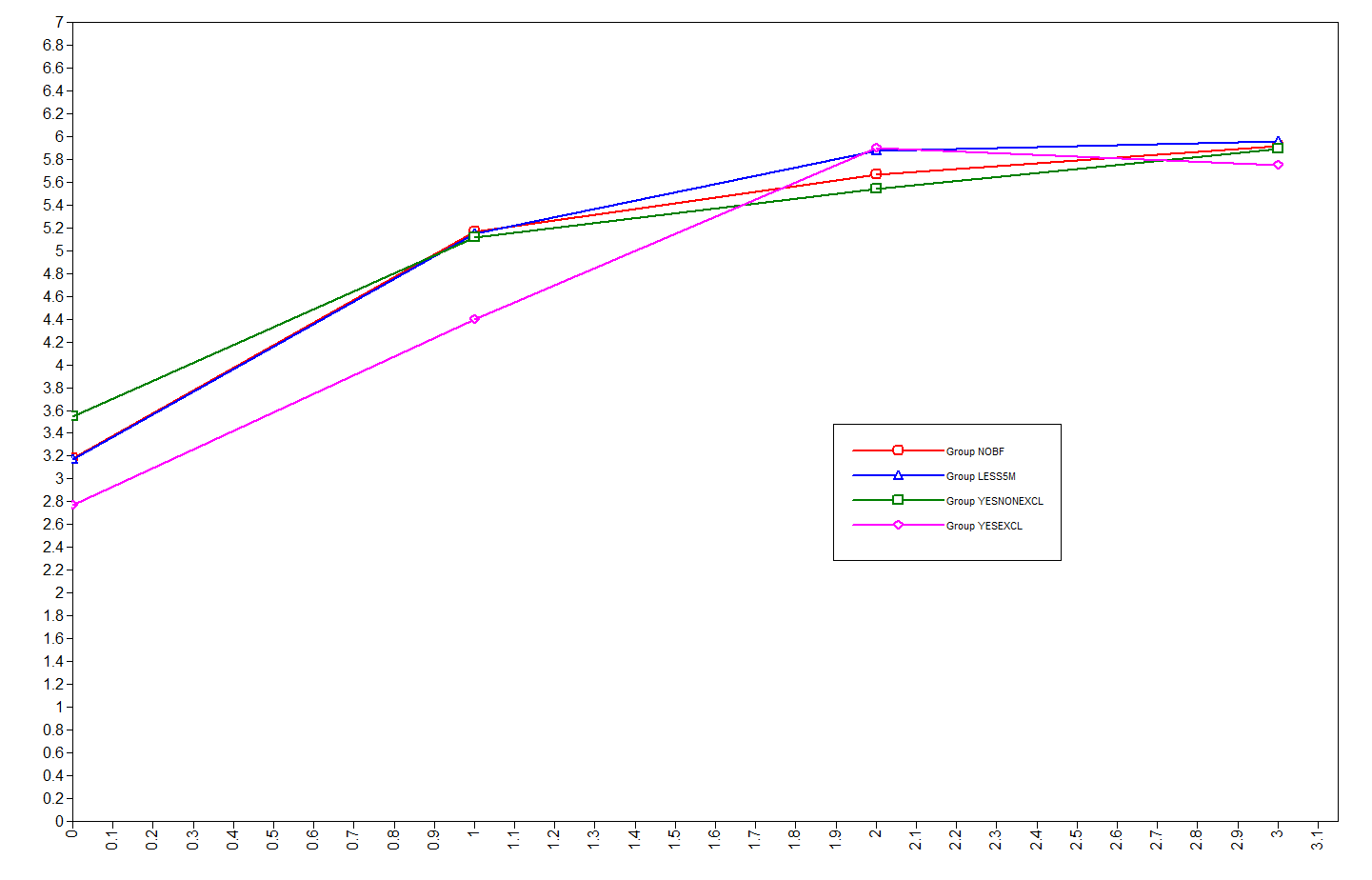


Figure S2. The figure displays the intercept and growth in memory-span from ages 4 to 7. The red line indicates non-breastfed children (n= 600, 28.3%). The blue line indicates children non-exclusively breastfed for 5 months or less (n= 809, 38.2%). The green line indicates children non-exclusively breastfed for more than 5 months (n= 356, 16.8%). The pink line indicates children exclusively breastfed for more than 5 months (n= 355, 16.7%). Data courtesy of the Quebec Institute of Statistics.
